# New onset severe right ventricular failure associated with COVID-19 in a young infant without previous heart disease

**DOI:** 10.1017/S1047951120001857

**Published:** 2020-06-16

**Authors:** Moises Rodriguez-Gonzalez, Patricia Rodríguez-Campoy, Maria Sánchez-Códez, Irene Gutiérrez-Rosa, Ana Castellano-Martinez, Amado Rodríguez-Benítez

**Affiliations:** 1 Division of Pediatric Cardiology, Puerta del Mar University Hospital, Cadiz, Spain; 2 Institute of Biomedical Research and Innovation of Cadiz (INIBICA), Research Institute, Puerta del Mar University Hospital, Cadiz, Spain; 3 Division of Pediatric Intensive Care Unit, Puerta del Mar University Hospital, Cadiz, Spain; 4 Division of Pediatric Infectious Diseases, Puerta del Mar University Hospital, Cadiz, Spain; 5 Division of Pediatric Nephrology, Puerta del Mar University Hospital, Cadiz, Spain; 6 Division of Radiology, Puerta del Mar University Hospital, Cadiz, Spain

**Keywords:** COVID-19, SARS-CoV-2, pulmonary hypertension, heart failure, cardiogenic shock, paediatric multisystem inflammatory syndrome

## Abstract

We present our recent experience with a 6-month-old infant with a personal history of short bowel syndrome that presented with fever, cyanosis, and cardiogenic shock secondary to severe pulmonary hypertension and right ventricular failure without pulmonary thromboembolism. He did not present signs of toxin-mediated disease or Kawasaki disease. He was finally diagnosed with SARS-CoV-2 infection. If this presentation is confirmed in future research, the severe cardiovascular impairment in children with COVID-19 could be also attributable to the primary pulmonary infection, not only to a multisystem inflammatory syndrome but also in children without heart disease.

COVID-19, caused by SARS-CoV-2, has different clinical features in paediatric and adult patients.^1^ In contrast to adults, children seem to be largely spared the severe cardiovascular involvement of SARS-CoV-2.^1^ Recently, the National Health Service England issued an alert regarding a small but growing number of paediatric cases with symptoms similar to those found in toxic shock syndrome and Kawasaki disease that required ICU admission with severe heart failure and myocardial dysfunction, which is now being labelled paediatric multisystem inflammatory syndrome.^2^ Therefore, there is an urgent need for the identification of children with COVID-19 infection and severe cardiovascular involvement in order to adequately characterise this association.

## Case presentation

**Figure 1.**
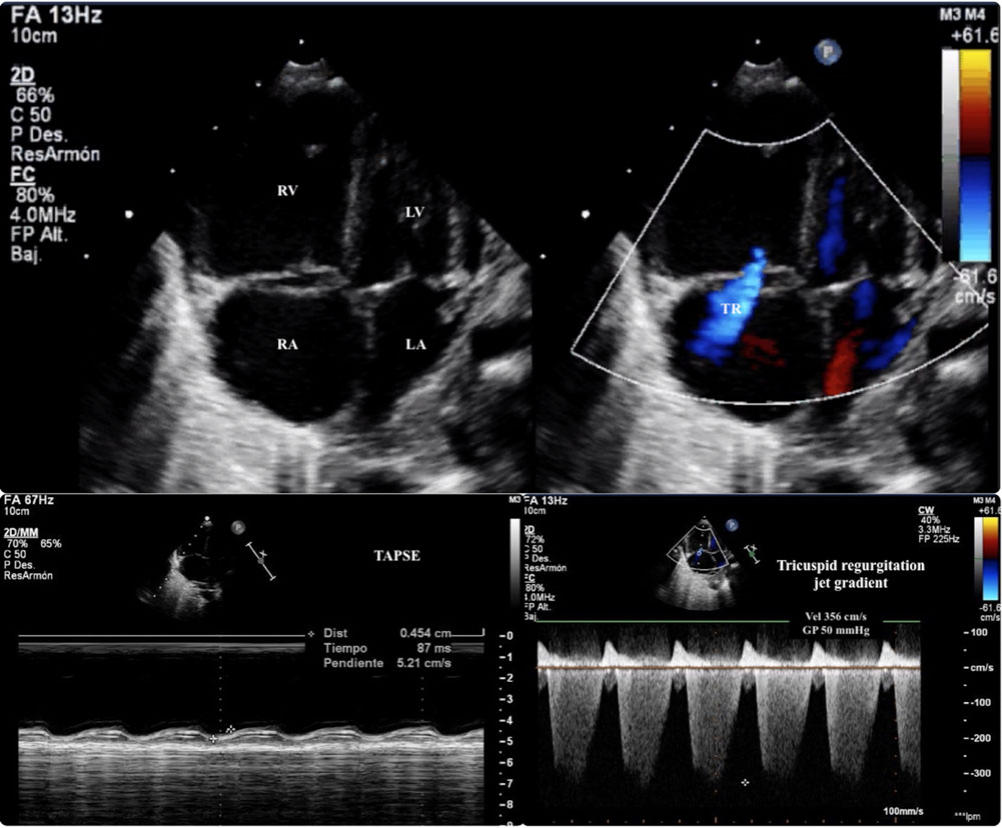
Echocardiography: The upper panel is an apical 4-chambers view that shows a dilated RV with severe tricuspid regurgitation and interauricular septum domed to the LA. The estimated systolic pulmonary arterial pressure that was 70 mmHg (50 mmHg of the TRJG + 20 mmHg of estimated RA pressure; the bottom right-sided panel), respect a systolic arterial pressure of 60 mmHg at the moment of the echocardiography. All the above constitute echocardiographic signs of supra-systemic PH). The bottom leftsided panel is a M-Mode in an apical 4-chambers view that shows a severely depressed RV systolic function (TAPSE 4.5 mm). RV (right ventricle); LV (left ventricle); RA (right atrium); LA (left atrium); TR (tricuspid regurgitation jet); TRJG (tricuspid regurgitation jet gradient); TAPSE (Tricuspid annular plane systolic excursion).

**Figure 2.**
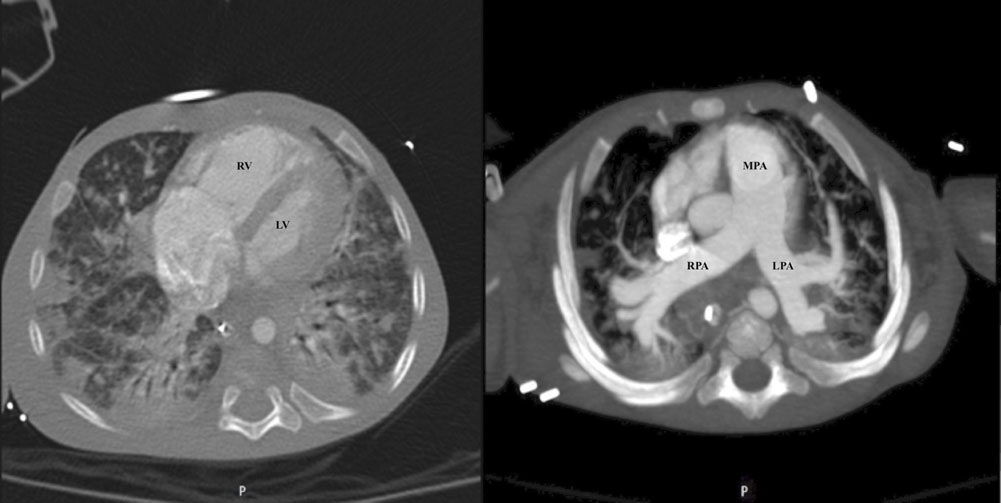
Thoracic angio-CT scan. The left panel shows multifocal regions of consolidation and ground-glass opacifications with peripheral and basal predominance. The right ventricle is severely dilated. No pleural or pericardial effusion. The right panel shows a severely dilated pulmonary artery with no signs of pulmonary thromboembolism (Maximum intensity projection reconstruction).RV (right ventricle); LV (Left ventricle); MPA (main pulmonary artery); LPA (left pulmonary artery); RPA (right pulmonary artery).

A 6-month-old male presented at our emergency department with severe respiratory distress and fever. He was diagnosed with short bowel syndrome secondary to multiple intestinal resections during the neonatal period. Echocardiographic studies during the neonatal period were unremarkable. He received domiciliary parenteral nutrition through a central tunnelled line at the superior vena cava. He was also under antithrombotic prophylaxis with low-molecular-weight heparin due to previous episodes of local thrombotic obstructions of the central line. Of note, the parents had forgotten to administer the heparin for 3 days since presentation. He presented with a 2-week history of nasal congestion and cough. During this period, two nasopharyngeal swab specimens for SARS-CoV-2 Real-time reverse-transcriptase polymerase chain reaction testing (Allplex™) were performed and were negative. The patient was immediately transferred to the paediatric ICU as he presented with irritability, tachypnoea (80 bpm), cyanosis (SpO_2_ 81%), tachycardia (170 bpm), hypotension (59/32 mmHg), poor perfusion, weak peripheral pulses, and hepatomegaly (3 cm). There were no other signs of toxin-mediated disease or Kawasaki disease, such as a cutaneous rash, conjunctivitis, diarrhoea, or lymphadenopathy. The major findings of the laboratory exams were haemoglobin 7 g/dl, haematocrit 24%, leukocytes 30.2 × 10^3^/µL (61% neutrophils, 29% lymphocytes, 0.9% eosinophils), platelets 98 × 10^3^/µL, ferritin 7634 ng/ml (22–275 ng/ml), C-reactive protein 86 mg/L (0.0–5.0 mg/L), procalcitonin 3.46 ng/L (<0.5 ng/L), D-Dimer 4200 ng/ml (0–500 ng/ml), prothrombin activity 63% (75–140%), fibrinogen 179 mg/dl (150–450 mg/dl), troponin I 90 ng/L (0.0–34.2 ng/L), NT-proBNP 26,000 pg/ml (0.0–125 pg/ml), interleukin-6 198 pg/ml (0.49–3.95 pg/ml), pH 7.10, pCO_2_ 62 cmH_2_O, and HCO_3_ 17 mmol/L. The lung ultrasound showed an irregular pleural line, B-lines, some coalescent, with bilateral patchy distribution, and small peripheral consolidations, which were larger in posterior-basal areas. The abdominal ultrasound was unremarkable, without signs of ascites or ileitis. The 12-lead electrocardiogram showed sinus tachycardia, right axis deviation, and right ventricular hypertrophy. Transthoracic echocardiography did not reveal any CHD with normal dimensions and systolic function of the left ventricle (left ventricular ejection fraction 62%). However, it showed severely dilated right chambers, severe right ventricular systolic dysfunction, and supra-systemic pulmonary hypertension (Fig 1). A thoracic angioCT-scan ruled out massive pulmonary thromboembolism but showed a pattern of ground glass and numerous consolidations of predominance in the posterior-basal segments of both lungs (Fig 2). Blood, urine, and stool cultures were sterile. A new SARS-CoV-2 Real-time reverse-transcriptase polymerase chain reaction test (Allplex™) from tracheobronchial secretions at ICU admission was negative. A Film-Array respiratory panel to detect other coronaviruses was also negative.

Mechanical ventilation was initiated in a prone position with a positive end-expiratory pressure of 12 cmH_2_O, a fraction of inspired oxygen of 1.0, and a tidal volume of 7 ml/kg. Inotropic support with milrinone (0.5 mcg/kg/min), norepinephrine (0.2 mcg/kg/min), and broad-spectrum antibiotics (meropenem, vancomycin, and fluconazole) were initiated. Despite no evidence of pulmonary thromboembolism, heparin was added at antithrombotic therapeutic dosages. The clinical state remained the same during the first 6 hours after the initiation of this treatment. Due to the high epidemiological, clinical, and radiological suspicion of SARS-CoV-2 infection, we decided to also initiate therapy with tocilizumab (12 mg/kg/12 h), azithromycin (5 mg/kg/d), hydroxychloroquine (7 mg/kg/d), and methylprednisolone (1 mg/kg/d). Three hours after starting this treatment (administration of 1 dose of each drug), we noticed a gradual and sustained respiratory and haemodynamic improvement, allowing a progressive reduction in respiratory and haemodynamic support. We performed a follow-up echocardiography after the administration of the third dose of tocilizumab, approximately 48 hours after ICU admission. The echocardiography revealed normal biventricular function and dimensions with normal pulmonary artery pressures. The patient remained afebrile, and there was complete normalisation of the cardiac and inflammatory biomarkers. Therefore, tocilizumab (administration of a total of three doses) and the inotropic support (approximately 24 hours of norepinephrine and 48 hours of milrinone infusion) were discontinued at this point. The patient completed 5 days of azithromycin, hydroxychloroquine, and methylprednisolone. No side effects of any drug were noticed.

He was extubated on day 4 and transferred to the paediatric ward on day 7 after ICU admission. SARS-CoV-2 was confirmed by the detection of anti-SARS-CoV-2 IgG (Abbott**®**; reported specificity 97.6%) on day 21 of illness, in the convalescence period after full recovery of the patient. He was finally diagnosed with cardiogenic shock secondary to severe pulmonary hypertension and new onset right ventricular failure associated with COVID-19.

## Discussion

We report a unique case of severe cardiovascular involvement associated with paediatric COVID-19. Our case highlights the fact that SARS-CoV-2 can also present in children as severe heart failure, even without previous heart disease. A concerning association between COVID-19 and the novel multisystem inflammatory syndrome has been recently noticed and increasingly reported.^2-4^ However, to the best of our knowledge, there are no similar cases of COVID-19 with new onset isolated right ventricular failure secondary to pulmonary hypertension attributable to the primary pulmonary infection in children.^5^ Our patient did not present any clinical signs of toxin-mediated disease, Kawasaki disease, or gastrointestinal findings that are typically present in multisystem inflammatory syndrome.^3,4^ Therefore, we think that our case should be differentiated from this novel entity.

Previous cases reported of severe pulmonary hypertension in adults were secondary to pulmonary thromboembolism,^6^ our first clinical suspicion due to the personal history of the patient. The pulmonary thromboembolism could not be demonstrated and the patient improved after the initiation of tocilizumab and steroids. Therefore, we hypothesise that the pulmonary endothelial cell damage leading to endothelitis, the activation of coagulation pathways, and deregulated inflammatory cell infiltration with cytokine storm could be the primary physiopathological mechanism of the severe pulmonary hypertension observed in our patient.^7,8^ At least some SARS-CoV-2-infected patients who become critically ill suffered a generalised thrombotic microvascular injury mediated by intense complement activation involving the lung.^9^

Based on our findings, the screening of myocardial dysfunction and pulmonary hypertension through cardiac biomarkers or echocardiography could be beneficial in severe COVID-19 paediatric cases, even if they do not fulfil case definition criteria for multisystem inflammatory syndrome. Failure to identify and accurately manage the severe myocardial dysfunction could worsen the prognosis of these patients. Furthermore, if similar cases with severe cardiovascular involvement are reported, children with CHD could be catalogued as a high-risk population for developing complications or COVID-19 disease of greater severity.

Finally, we noticed a rapid improvement after the empirical initiation of some drugs used to treat COVID-19 in adults. If those drugs were shown to be effective and safe therapies against SARS-CoV2 infection in children, we would encourage their use when there is a high clinical suspicion, even for patients with previous negative Real-time reverse-transcriptase polymerase chain reaction tests, since the accuracy of current microbial assays for SARS-CoV-2 is still modest and variable.^10^
